# COVID-19 outbreak in Jordan: Epidemiological features, clinical characteristics, and laboratory findings

**DOI:** 10.1016/j.amsu.2020.07.020

**Published:** 2020-07-18

**Authors:** Shaher M. Samrah, Abdel-Hameed W Al-Mistarehi, Ali M. Ibnian, Liqaa A. Raffee, Suleiman M. Momany, Musa Al-Ali, Wail A. Hayajneh, Dawood H. Yusef, Samah M. Awad, Basheer Y. Khassawneh

**Affiliations:** a Department of Internal Medicine, Faculty of Medicine, Jordan University of Science and Technology (JUST), Irbid, Jordan; b Department of Public Health and Family Medicine, Faculty of Medicine, Jordan University of Science and Technology (JUST), Irbid, Jordan; c Department of Accident and Emergency Medicine, Faculty of Medicine, Jordan University of Science and Technology (JUST), Irbid, Jordan; d Department of Pediatrics and Neonatology, Faculty of Medicine, Jordan University of Science and Technology (JUST), Irbid, Jordan

**Keywords:** SARS-CoV-2, COVID-19, Coronavirus, c-Reactive protein, Erythrocyte sedimentation rate, BCG, D-dimer, Outbreak, Jordan

## Abstract

**Background:**

In March 2020, an outbreak of coronavirus 19 (COVID-19) was detected in the North of Jordan. This retrospective study is the first from Jordan to report the epidemiologic, clinical, laboratory, and radiologic characteristics of COVID-19 infected patients.

**Methods:**

All patients with laboratory-confirmed COVID-19 infection by RT-PCR in the North of Jordan admitted between March 15 and April 2, 2020 were included. The clinical features, radiological, and laboratory findings were reviewed.

**Results:**

Of 81 patients affected, 79 (97.5%) shared a common exposure to four recent travelers from endemic areas. The mean age was 40 years. Although about half (44 [54.3%]) were females, symptomatic patients were mostly females (75%). The most common presenting symptoms were nasal congestion, sore throat and dry cough. Less than one-third (31%) had chronic diseases. Although 84% of patients reported receiving Bacille Calmette-Guérin (BCG) vaccination, more asymptomatic patients had BCG than symptomatic (*p* = 0.017). Almost all patients (97.5%) had an elevated D-dimer level. Erythrocyte sedimentation rate (ESR) and c-reactive protein were elevated in 50% and 42.7% of patients, respectively. High ESR found to be the predictor of abnormal chest radiograph observed in 13 (16%) patients with OR of 14.26 (95% CI 1.37–147.97, *p* = 0.026).

**Conclusions:**

An outbreak of COVID-19 infection in northern Jordan affected more females and relatively young individuals and caused mainly mild illnesses. The strict outbreak response measures applied at early stages probably contributed to the lenient nature of this outbreak, but the contribution of other factors to such variability in COVID-19 presentation is yet to be explained.

## Highlights

• A COVID-19 outbreak in northern Jordan caused mild illness presentation and most cases shared a common exposure.
• Young individuals and females were mainly affected in this outbreak.
• The majority and more of the asymptomatic patients, reported receiving BCG immunization.
• Inflammatory markers were elevated in most cases and Elevated ESR was a predictor to abnormal chest radiograph findings.
• Early and strict response measures limited COVID-19 spread in Jordan and contributed to the lenient nature of the outbreak.

## 1 Introduction

In December 2019, China reported to the World Health Organization (WHO) multiple cases of unexplained lower respiratory tract infections. A coronavirus that is genetically related to the SARS virus was identified (SARS-CoV-2). This new viral syndrome named by the WHO as coronavirus disease 2019 (COVID-19). The epidemic spread within mainland China with a basic reproduction number (R_0_) estimated to be from 2.2 to 3.3 and a mortality rate of around 2.3% [[1], [2], [3]]. Recent studies in China have found that patients infected with COVID-19 were mostly above 30 years old and mainly males. Common presenting symptoms include fever, dry cough, and fatigue. Also, laboratory abnormalities were mainly lymphopenia [[4], [5], [6], [7]]. In a study from Singapore, mild respiratory tract infection was the main complaint in patients with COVID-19, and few patients required supplemental oxygen [8].

Epidemiological and clinical data of patients infected with COVID-19 in the Middle East region have not been reported. This study aims to describe the epidemiologic characteristics, relevant clinical, laboratory and radiological features of patients with laboratory-confirmed SARS-CoV-2 infection during the COVID-19 outbreak in the North of Jordan.

## 2 Methods

### 2.1 Outbreak response

Jordan is a Middle Eastern country with a population of 11 million. After the WHO declared COVID-19 as pandemic disease, the Jordanian Ministry of Health issued a health-alert that patients with flu-like symptoms and recent travel to endemic countries should be screened for SARS- CoV-2 infection. After the first case with COVID-19 infection was reported in Jordan on March 2, 2020, a policy of extensive contact tracing followed by quarantine of asymptomatic contacts, and hospital isolation and screening of symptomatic contacts were placed. This was supported by a countrywide strict lockdown and a nightly curfew. The borders were sealed off and all traveling to and from Jordan was stopped on March 15, 2020. A Royal Decree has been issued approving the implementation of the National Defense Law on March 17, 2020. These robust measures were successful in flattening the curve of the spread of COVID-19 infection and reducing the public health impact.

### 2.2 Participants, study Design, specimen, and data collection

King Abdullah University Hospital (KAUH) was assigned by the ministry of health to provide medical care for patients with COVID-19 infection in the North of Jordan. The hospital serves a population exceeding 2 million. All adult patients (≥18 years) who were admitted to KAUH from March 15 to April 2, 2020 and had a laboratory-confirmed COVID-19 infection, were included in this study. All cases with symptoms of upper or lower respiratory tract infection, with a recent contact with a diagnosed COVID-19 case, or those who underwent a random screening regardless of their symptoms were tested for COVID-19 via a nasopharyngeal swab. A confirmed case of COVID-19 was defined by a positive SARS-CoV-2 real-time reverse transcriptase-polymerase chain reaction assay (RT-PCR), from a collected nasopharyngeal swab specimen. Specimens were collected and analyzed according to the Centers for Disease Control and Prevention (CDC) guidelines. All clinical specimens were tested with the assay developed by the CDC, targeting the N1 and N2 genes [9].

Clinical charts, nursing records, laboratory findings, and chest x-rays of all patients with laboratory-confirmed COVID-19 infection were retrospectively reviewed. Demographic data, medical and exposure history, underlying comorbidities, laboratory results, and radiological findings were extracted. Data, that was not available in the medical records, was obtained through direct communication with patients or their families. Bacille Calmette-Guérin (BCG) vaccination status was obtained from the patient and confirmed by identifying the skin scar/mark on the left upper arm, the usual site for the BCG vaccine. Patients who denied having the vaccine and/or did not have the scar/mark were considered as not vaccinated.

Informed consent was obtained from the recruited patients. All procedures performed in this study involving human participants were reviewed and ethically approved by the Institutional Review Board (IRB) and research and ethics committee at Jordan University of Science and Technology. This study was conducted following the 1975 Helsinki declaration, as revised in 2008 and its later amendments or comparable ethical standards [10]. This work has been reporting based on STROCSS 2019 guidelines (Strengthening the Reporting of cohort studies in surgery) [11]. The protocol had been registered at research Registery with the unique identification number researchregistry5728 [12].

### 2.3 Statistical analysis

The characteristics of patients were described using frequency and percentage for categorical variables and mean ± standard deviation for continuous variables. A chi-square test or Fisher's exact test was used to assess the association between categorical variables, whereas continuous variables were analyzed by the Student's t-test or ANOVA. A binary logistic regression analysis was conducted to determine the predictors of abnormal chest radiographs including age, obesity, presence of comorbidities, and inflammatory markers (ESR and CRP). Odds ratio (OR) and their 95% confidence intervals (95% CI) were reported. A *p*-value of less than 0.05 was considered statistically significant. The IBM Statistical Package for Social Sciences Software (SPSS) for Windows, version 25.0 was used for data processing and analysis.

## 3 Results

After the outbreak was recognized on March 17th, 2020, a total of 81 adult patients were admitted with COVID-19 infection by April 2, 2020. The vast majority (79 patients) shared a common exposure to an index case who had recent travel from an endemic area [13]. Two patients were from the nursing staff at KAUH. The majority of patients were relatively young; 43 (53.1%) were aged 18–39 years, 32 (39.5%) were aged 40–64 and 6 (7.4%) were older than 64 years. The mean ± SD age of the patients was 40.0 ± 16.6 years (range 18–80) and 54.3% were females. About one third were cigarette smokers, 35.5% were obese (BMI ≥ 30 kg/m2), and 31% had chronic illnesses such as ischemic heart disease, hypertension, dyslipidemia, diabetes mellitus, and malignancy. Four female patients were pregnant. The majority of the patients (84%) received BCG vaccination. The demographic and clinical characteristics are shown in Table 1.

**Table 1:** Demographics and clinical characteristics at the time of admission.

	All patients (n = 81)
Age, m±SD	39.95 ± 16.59
Female gender	44 (54.3%)
**Comorbidities**	25 (30.9%)
Diabetes Mellitus	10 (12.3%)
Hypertension	17 (21.0%)
Cardiovascular disease	6 (7.4%)
Dyslipidemia	7 (8.6%)
Malignancy	1 (1.2%)
Pregnancy	4 (4.9%)
History of travel from endemic area within 4 weeks	4 (4.9%)
History of contact with a confirmed case	77 (95.1%)
BCG vaccination	68 (84%)
**Vital signs**, (m±SD)	
Temperature (°C)	37.27 ± 0.55
Pulse oximeter O2 saturation %	95.63 ± 2.21
SpO2 90–94%	13 (16%)
SpO2 ≤ 89%	2 (2.5%)
Heart Rate/min	90.85 ± 14.59
Respiratory rate/min	19.42 ± 1.49
Systolic blood pressure (mmHg)	127.05 ± 15.48
Diastolic blood pressure (mmHg)	77.47 ± 8.36

**Fig. 1:**
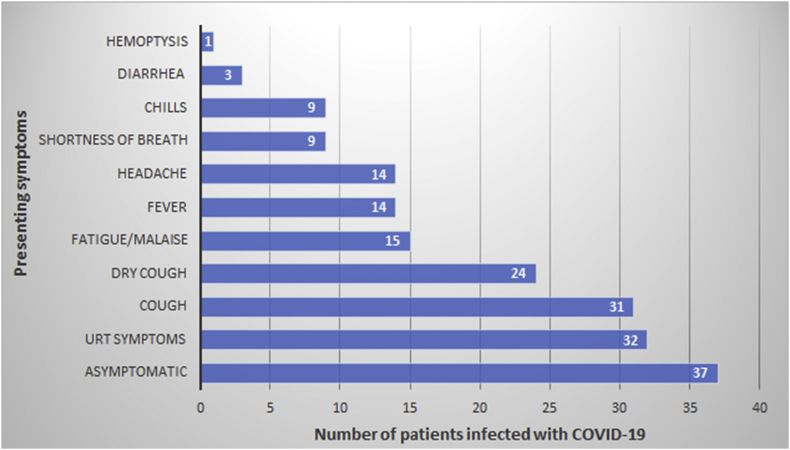
Presenting symptoms of patients with laboratory-confirmed COVID-19.

On admission about half of the patients (45.7%) were asymptomatic. The most common presenting symptoms in the symptomatic patients were upper respiratory tract symptoms, manifested as sore throat, and/or nasal congestion (40%), followed by dry cough (29%), malaise (19%) and fever (17%). Less common symptoms were headache (17.3%), shortness of breath (11%), chills/rigors (11%), productive cough (9%), diarrhea (4%), and hemoptysis (1%), **(**Fig. 1**)**. Only two patients had an oxygen saturation of less than 90%. None of the patients presented with severe pneumonia requiring admission to the intensive care unit. Leucocytes count were within the normal range (4–11 x 103/mm3) in 64 (79%) patients and leukopenia was reported in 10 (12.3%) patients. Neutropenia and lymphopenia were seen in 3 (3.7%) and 8 (9.9%) of the patients, respectively. Platelets were below the normal range in 8 (9.9%) patients and 15 (18.5%) patients had liver function abnormality. C-reactive protein (CRP) was elevated in 32/75 (42.7%) and erythrocyte sedimentation rate (ESR) was increased in 35/70 (50%). D-dimer was positive (>0.5 mg/l) in 97.4% of the patients. A chest x-ray on admission was done in 77 patients; 12 had patchy infiltrates and one patient had lobar infiltrate, **(**Table 2**)**.

**Table 2:** Laboratory and radiographic findings on admission to the hospital.

Parameters	All patients (n = 81)
White cell count x 10^3^/mm^3^, m±SD	7.00 ± 2.58
White cell count < 4 × 10^3^/mm^3^	10 (12.3%)
White cell count > 11 × 10^3^/mm^3^	7 (8.6%)
Neutrophils count x 10^3^/mm^3^, m±SD	4.37 ± 2.22
Neutrophils count >7000/mm^3^	8 (9.9%)
Neutrophils count <1500/mm^3^	3 (3.7%)
Lymphocytes count x 10^3^/mm^3^, m±SD	1.95 ± 0.81
Lymphocytes count >3000/mm^3^	8 (9.9%)
Lymphocytes count <1000/mm^3^	8 (9.9%)
Hemoglobin g/dl, m±SD	13.86 ± 2.04
Low Hb < 13 g/dl	22 (27.2%)
Platelets x 10^3^/mm^3^, m±SD	229.42 ± 67.53
Serum creatinine μmol/l, m±SD	69.51 ± 18.77
ALT u/l, m±SD	21.87 ± 15.66
AST u/l, m±SD	21.40 ± 7.65
Serum albumin g/l, m±SD	44.93 ± 3.96
CRP mg/l, m±SD (n = 75)	10.86 ± 24.49
High CRP (>5 mg/l)	32/75 (42.7%)
ESR mm/h, m±SD (n = 70)	22.53 ± 16.49
High ESR (>20 mm/h)	35/70 (50.0%)
D-dimer μg/ml, m±SD (n = 78)	1.05 ± 0.44
Positive D-dimer (>0.5 μg/ml)	76/78 (97.4%)
Abnormal chest radiographic findings	13/77 (16.9%)
Bilateral patchy infiltrates	7 (53.8%)
Unilateral patchy infiltrates	6 (46.2%)

ALT: alanine aminotransferase, AST: aspartate aminotransferase, CRP: C-reactive protein, ESR: erythrocyte sedimentation rate.

In comparison to asymptomatic patients, symptomatic patients were mostly females (75%), with an unadjusted odds ratio (OR) of 7.09 (95% CI 2.66–18.92, *p* < 0.001). Also, symptomatic patients were found to have higher ESR values, with OR of 6.77 (95% CI 2.31–19.84, *p* < 0.001). Although that the majority of patients received BCG vaccination, more asymptomatic patients (35/37, 94.6%) received the vaccination than symptomatic ones (33/44, 75%) with an OR of 5.83 (95% CI 1.20–28.32, *p* = 0.017). Chest x-ray was abnormal in 10/40 (25.0%) of symptomatic patients compared to 3/37 (8.1%) of those who are asymptomatic, (*p* = 0.048). There was no significant difference in age, smoking status, obesity, comorbidities, and other laboratory results between symptomatic and asymptomatic patients, **(**Table 3**)**.

**Table 3:** Demographic and laboratory findings according to the presenting symptoms.

	Symptomatic (n = 44)	Asymptomatic (n = 37)	*P* value
Age, m±SD	40.57 ± 15.67	39.22 ± 17.81	0.400
Female gender	33 (75%)	11 (29.7%)	<0.001
Cigarettes smoking	11 (25.0%)	15 (40.5%)	0.136
Obese (BMI ≥ 30 kg/m^2^)	14 (34.1%)	13 (37.1%)	0.786
Comorbidities	13 (29.5%)	12 (32.4%)	0.779
BCG vaccination	33 (75%)	35 (94.6%)	0.017
Neutropenia	3 (6.8%)	0	0.246
Lymphocytopenia	6 (13.6%)	2 (5.4%)	0.279
Abnormal Chest radiograph	10 (25.0%)	3 (8.1%)	0.048
CRP, mg/l, m±SD	14.67 ± 31.45	5.74 ± 6.65	0.119
High CRP (>5 mg/l)	21 (48.8%)	11 (34.4%)	0.210
ESR, mm/h, m±SD	27.80 ± 15.26	15.10 ± 15.45	0.001
High ESR (>20 mm/h)	28 (68.3%)	7 (24.1%)	0.001
D-dimer, μg/ml, m±SD	1.11 ± 0.52	0.97 ± 0.30	0.165
Positive D-dimer (>0.5 μg/ml)	42 (100%)	34 (94.4%)	0.122

Abnormal chest radiographic findings were seen more in patients older than 50 years, obese patients, and those with higher acute phase reactants (CRP, and ESR), **(**Table 4**)**. Using binary logistic regression while adjusting for confounding factors including age, obesity, presence of comorbidities, high CRP, and high ESR; only high ESR was associated with abnormal chest radiograph; adjusted odds ratio (OR) of 14.26 (95% CI 1.37–147.97, *p* = 0.026), **(**Table 5**).**

**Table 4:** Demographic and laboratory findings according to the chest radiographic findings.

Age, m±SD	Chest Radiographic Findings		*P* value
	Normal (n = 64)	Abnormal (n = 13)	
	37.78 ± 16.47	51.08 ± 14.07	0.008
Female gender	32 (50%)	8 (61.5%)	0.448
Cigarettes smoking	22 (34.4%)	4 (30.4%)	0.802
Obese (BMI ≥ 30 kg/m^2^)	17 (27.9%)	8 (72.7%)	0.004
Comorbidities	17 (26.6%)	7 (53.8%)	0.053
BCG vaccination	56 (87.5%)	10 (76.9%)	0.320
Neutropenia	2 (3.1%)	1 (7.7%)	0.430
Lymphocytopenia	5 (7.8%)	3 (23.1%)	0.128
Cough	21 (32.8%)	7 (53.8%)	0.151
Shortness of breath	5 (7.8%)	3 (23.1%)	0.128
Documented fever	9 (14.1%)	4 (30.8%)	0.143
High CRP (>5 mg/dl)	17 (29.3%)	11 (84.6%)	<0.001
High ESR (>20 mm/h)	21 (38.9%)	11 (91.7%)	0.0014
Positive D-dimer (>0.5 μg/ml)	62 (98.4%)	11 (91.7%)	0.184

**Table 5:** Predictors of abnormal chest radiology.

Variable	Odds Ratio (95% C.I.)	*P* value
Age	1.025 (0.963–1.091)	0.439
Obese (BMI ≥ 30 kg/m^2^)	3.238 (0.538–19.487)	0.199
Comorbidities	1.003 (0.122–8.226)	0.998
High CRP	4.898 (0.722–33.231)	0.104
High ESR	14.256 (1.374–147.967)	0.026

## 4 Discussion

This is the first study from the Middle East area to describe clinical and epidemiologic features, laboratory and imaging aspects, of patients with COVID-19 infection. Women were more affected than men and the majority of patients were relatively young. Moreover, the majority of patients were either asymptomatic or had mild disease. Increased inflammatory markers were common among symptomatic patients and elevated ESR was a predictor of abnormal chest radiograph.

In this study, only 12.4% of the patients were older than 60 years, which is lower than what has been described in Asia and Europe [[4], [5], [6], [7], [8]]. This reflects the demographic nature of the Jordanian population; 5.5% of the population is ≥ 60 years old and 62.9% < 30 years old [14].

All patients presented in our study had either mild or no symptoms. Mild upper respiratory tract infection symptoms were the most common presenting symptoms followed by a dry cough. Fever was an uncommon presenting symptom. Besides, females were significantly more symptomatic than males, unlike what has been reported in the literature [15,16].

In regard to the laboratory findings, CRP and ESR were elevated in 43% and 50% of patients, respectively but ESR was significantly higher in symptomatic patients. Although the majority of our patients had a mild disease presentation, almost all patients tested for D-dimer were found to have values above the locally defined cut-off value of >0.5 μg/ml. Tang et al. studied 183 cases with COVID-19 infection and found nearly 3.5 fold higher D-dimer values in patients with severe disease [17].

Many studies reported normal chest radiographs in early or mild disease. In a retrospective study of 64 patients in Hong Kong, 20% of patients did not have any abnormalities on chest radiographs at any point during the illness [18]. In our study, 17% had infiltrates in chest radiographs and were significantly more common in symptomatic patients. Obese patients, patients >50 years old, and those with elevated ESR and CRP had a higher incidence of lung infiltrates. Patients with comorbidities such as Diabetes Mellitus and hypertension had a higher incidence of abnormal chest radiograph but without statistical significance (*p* = 0.053).

Several studies described varying degrees of illness and severity: mild, severe, or critical [19,20]. In Singapore, among the first 18 cases of COIVD-19, mild respiratory tract infection was the most common presentation with some patients requiring supplemental oxygen [8]. A study from the Chinese Center for Disease Control and Prevention with more than 44,000 confirmed cases has reported no or mild pneumonia in 81%, while 19% were considered severe or critical with an overall case fatality rate (CFR) of 2.3% [19]. CFR reported worldwide is variable, probably related to the population demographic features (such as age distribution of the population), with as high as 5.8% in Italy [21] to 0.7% in South Korea [22].

Our results showed a mild spectrum of severity in the clinical presentation of this outbreak. Similar presentations have been described as an initial trend of COVID-19 outbreak presentation in countries like Singapore and New Delhi [6,8,[23], [24], [25]], **(**Table 6**)**. The mildness of this outbreak might be explained by the including of asymptomatic cases and the fact that the majority of our cases (97.5%) shared a common source of exposure, an index case who lives in Spain and traveled to Jordan which resulted in a local outbreak of COVID-19 [13]. Sharing the same COVID-19 strain by most of our cases at almost the same period might have produced a similar and comparable mild presentation seen in the majority of our cases. This observation may not explain a similar mild spectrum of disease and lower CFR observed at the national level of Jordan as well. Although it is too early to have an accurate mortality rate of COVID-19 infection, as there is no accurate information of the true number of infected cases, the estimated national Jordanian CFR as of April 18, 2020, is around 1.7% (7/407). This appears to be lower than the estimated global CFR of around 6.8% (154,726 cases/2,261,037 confirmed cases).

**Table 6:** Comparison with other initial studies from different countries.

	Current study	Gupta et al. [23]	Chen et al. [6]	Young et al. [8]	Saleemi et al. [24]	Khamis et al. [25]
Location of study	Jordan	New Delhi, India	Wuhan, China	Singapore	Saudi Arabia	Oman
Setting	Hospitalized patients	Hospitalized patients	Hospitalized patients	Hospitalized patients	Hospitalized patients	Hospitalized patients
Number of cases	81 (2 severe)	21 (one severe)	99 (17 severe)	18 (6 severe)	51 (21 severe)	63 (24 severe)
Age, years	40 (mean) 18 - 39 (range)	40.3 (mean) 16 - 73 (range)	55·5 (mean) 21 - 82 (range)	47 (median) 31 - 73 (range)	49 (median) 30, 66 (Q1, Q3)	48 (mean) 22 - 87 (range)
Males, n (%)	37 (45.7%)	14 (66.7%)	67 (67.7%)	9 (50%)	23 (45%)	53 (84%)
Comorbidities, n (%)	25 (30.9%)	6 (28.6%)	50 (51%)	5 (28%)	–	32 (51%)
Asymptomatic, n (%)	37 (45.7%)	9 (42.9%)	–	–	–	–
The most common presenting symptoms, n (%)	URT symptoms, 32 (40%); Cough, 31 (29%); Fatigue, 15 (19%); Fever, 14 (17%)	Fever, 9 (42.9%); Cough, 9 (42.9%); Sore throat, 5 (23.8%)	Fever, 82 (83%); Cough, 81 (82%); Dyspnea, 31 (31%)	Cough, 15 (83%); Fever, 13 (72%); Sore throat, 11 (61%)	Cough (69%); Fatigue (67%); Fever (63%)	Fever, 53 (84%); Cough, 47 (75%); Dyspnea, 37 (59%)
Laboratory data						
WBCs	↑8.6%; ↓12.3%	↓4.8%	↑24%; ↓9%	–	–	↑16%; ↓11%
Neutrophils	↑9.9%; ↓3.7%	–	↑38%	–	–	–
Lymphocytes	↑9.9%; ↓9.9%	–	↓35%	↓7 of 16 (38.9%)	–	↑3.2%; ↓47%
CRP	↑42.7%	–	↑86%	↑6 of 16 (38%)	–	↑92%
ESR	↑50.0%	–	↑85%	–	–	–
D-dimer	↑97.4%	–	↑36%	–	–	↑59%
Radiological findings	7 (53.8%) had bilateral patchy infiltrates, and 6 (46.2%) had unilateral patchy infiltrates in the lower lobes of lungs	one patient showed bilateral consolidation of lower lobes of lungs	74 (75%) had bilateral pneumonia, 14 (14%) showed multiple mottling and ground-glass opacity, and one (1%) had pneumothorax	6 (33%) had an abnormal chest radiograph finding or lung crepitations.	6 (12%) had focal and 22 (43%) bilateral opacities	15 (24%) had Major bilateral abnormality, and 45 (73%) showed Infiltrations/patchy shadowing
Mortality rate, n (%)	One (1.2%)	Zero	11 (11%)	Zero	2 (3.9%)	5 (8%)

Laboratory data are reported as percent of patients with abnormalities defined according to the local reference ranges. URT symptoms, Upper respiratory tract symptoms, manifested as sore throat, and/or nasal congestion; WBCs, White blood cells count; CRP, C-reactive protein; ESR, erythrocyte sedimentation rate.

There is no clear evidence that BCG immunization has a positive impact on COVID-19 morbidity and mortality at the present time [26]. Two studies have suggested that BCG immunization, given routinely after birth and at school age in countries with a higher incidence of tuberculosis (TB) to primarily prevent TB meningitis, induces a nonspecific-immune response that may have protective effects against viral infections [27,28]. Although few studies suggested a trend toward a protective effect of BCG vaccination, these studies were prone to significant bias from many variables, including differences in national demographics and disease burden, testing rates for coronavirus infections, and the stage of the pandemic in each country [[29], [30], [31]]. BCG vaccination policy is adopted as part of the national immunization program of Jordan in 1970. Since 1983, children with no vaccination scar were vaccinated at school age. Although patients who received BCG immunization in our study were found to have a higher chance of being asymptomatic, the small cohort size and cluster nature of our study population cannot be representative to conclude a protective effect of BCG immunization on the severity or lower incidence of COVID-19 infection. World Health Organization (WHO) recommends BCG vaccination not to be used for prevention or lessening the severity of COVID-19, pending further data [32].

The outbreak response measures, that were adopted and strictly applied by the government, in addition to the fact that COVID-19 has affected younger population probably have contributed to the lower incidence as well as lower morbidity and mortality of COVID-19 infection in Jordan. Since the early reports of COVID-19 cases, the Jordanian government promptly applied a strict lockdown to prevent further spread of the infection. On March 17, with 29 COVID-19 confirmed cases, a lockdown was announced, which turned into a strictly enforced curfew that was described as one of the world's strictest measures. Upon observing the early signs of the local outbreak, the government issued an isolation order of the northern district, distinctly to the governorate of Irbid. Further measures were followed, starting by extensive contact tracing and strict isolation of all affected buildings and local neighborhoods, ending by sealing off the national air, sea and land borders. In contrast, applying similar measures at a later stage of COVID-19 outbreak by other countries appeared to be less effective in controlling the spread of the infection. Although applying such measures at earlier stages of the outbreak was considered by some as relatively extreme, it appears to have helped limiting the viral spread and probably contributed to the mildness of the presentation of our affected population. While studies are being conducted to determine the benefits of several drugs against COVID-19 such as antiviral protease inhibitors, and immunosuppressants [33,34], we recommend developing strict guidelines for isolation, and triage of infected COVID-19 patients as well as stringently applyingoutbreak response measures as early as possible.

Our study has several notable limitations including the small cohort size. First, most of our cases were mild cases with limited exposure to a critically ill spectrum of disease. Second, BCG immunization was obtained by questioning the patients rather than having documented medical records as most were patients who were seen for the first time at our hospital. Third, some cases had incomplete documentation of clinical symptoms and were missing laboratory testing or both. However, as this is the first observational study from the Middle East area, it increases the awareness of a different presentation of COVID-19 infection in the area and provide a better understanding of various factors that might be contributing to the milder nature of disease severity.

## 5 Conclusion

In the northern Jordan region, the majority of COVID-19 outbreak shared a common source of exposure. Most of the cases were distinctively in the milder spectrum of disease severity with most of the patients were either asymptomatic or had mild disease. The infection affected more females and relatively young individuals. Despite the fact that the presentation of this outbreak appears to be mild, D-dimer was elevated in almost all patients. Additionally, inflammatory markers seem to play a role in predicting disease severity. The limited contact exposure and strict outbreak response measures probably contributed to the lenient nature of this outbreak, but the contribution of other factors such as BCG-immunization to such a variability in COVID-19 presentation seems to dictate further investigation.

## Compliance with ethical standards

All procedures performed in this study involving human participants were reviewed and ethically approved by the Institutional Review Board (IRB) and research and ethics committee at Jordan University of Science and Technology. This study was conducted following the 1975 Helsinki declaration, as revised in 2008 and its later amendments or comparable ethical standards. This work has been reporting based on STROCSS 2019 guidelines (Strengthening the Reporting of cohort studies in surgery).

## Informed consent

Informed consent was obtained from all individual participants included in the study.

## Research registration unique identifying number (UIN)

Name of the registry: Research Registery.

Unique Identifying number or registration ID: researchregistry5728.

Hyperlink to the specific registration:

https://www.researchregistry.com/browse-the-registry#home/registrationdetails/5eebd7e72266490015e93a21/.

## Availability of data and materials

The datasets generated and analyzed during the current study are available with the corresponding author.

## Funding

No Funding was received for this study.

## Ethical approval

Institutional approval was obtained from the Institutional Review Board at Jordan University of Science and Technology.

## Consent

Written informed consent was waived due to the retrospective nature of the study.

## Author contribution

All authors contributed significantly and in agreement with the content of the article. All authors were involved in project design, data collection, analysis, statistical analysis, data interpretation and writing the manuscript. All authors presented substantial contributions to the article and participated of correction and final approval of the version to be submitted.

## Guarantor

Shaher Samrah.

## Provenance and peer review

Not commissioned, externally peer reviewed.

## Declaration of competing interest

The authors declare that they have no competing interests.

## Appendix A Supplementary data

Multimedia component 1 Multimedia component
1
